# Ventilation and Fresh Air

**Published:** 1882-04

**Authors:** 


### VENTILATION AND FRESH AIR.

COMFORTS of the fireside are almost inseparable from the idea of an open fireplace, and from a hygienic standpoint, too, the old-fashioned chimney, or an open grate, is far superior to a closed stove. But it should not be forgotten, writes Dr. Oswald in the *Popular Science Monthly*, that the operation of the chimney-draft alone is insufficient to correct the vitiated air of a small room; it merely creates an outward current. An open window completes the renovating process; in cold weather a few minutes are sufficient to revitalize the indoor atmosphere for a couple of hours. Only the blindest prejudice can deny the pleasant effect of such an influx of life-air; it revives the azotized lungs as a draft of cool water refreshes the parched palate. Colds are never caught in that way. The very name is a misleading misnomer; inflection or influenza would be the right word. Long exposure to a freezing storm, in certain cases, induces a pure pleuritic fever—a very rare affection, and entirely different from the only too familiar catarrh.

What we call a cold is caused by the influence of impure air, or dust, on the sensitive tissue of our respiratory organs; subsequent exposure to the open air merely intimates the crisis of the disorder—the discharge of the accumulated mucus through the nose or throat. Fresh air is here only the proximate cause, as in toothache, or in those paroxysms of retching following upon the first respiration of a half-drowned person. If we postpone the crisis by persistently avoiding the open air, the unrespirable matter, instead of being discharged, will be deposited in the tissue of the lungs in the form of tubercles.

A LAUGH is not always the symbol of a frank character. You meet in this world with false mirth as often as with false gravity; the grinning hypocrite is not a more uncommon character than the groaning one.

THE only safe and immediate remedy within the reach of a non-professional, in case of poisoning with prussic acid, is to pour a stream of cold water from an elevation upon the head and spine of the patient.

